# Pericytes Are Immunoregulatory Cells in Glioma Genesis and Progression

**DOI:** 10.3390/ijms25105072

**Published:** 2024-05-07

**Authors:** Marta Martinez-Morga, Daniel Garrigos, Elena Rodriguez-Montero, Ana Pombero, Raquel Garcia-Lopez, Salvador Martinez

**Affiliations:** 1 Instituto de Neurociencias, Universidad Miguel Hernández–CSIC, Excellence Center Severo Ochoa, Campus de San Juan, Avda. Ramón y Cajal sn, 03550 Alicante, Spain; marta.martinezm@umh.es (M.M.-M.); dgarrigos@umh.es (D.G.); elenarodriguezmontero@gmail.com (E.R.-M.); apombero@umh.es (A.P.); r.garlo@umh.es (R.G.-L.); 2 Centro de Investigación Biomédica en Red en Salud Mental, CIBERSAM-ISCIII, 46010 Valencia, Spain

**Keywords:** glioma immunotolerance, vascular co-option, tumor microenvironment, perivascular cells

## Abstract

Vascular co-option is a consequence of the direct interaction between perivascular cells, known as pericytes (PCs), and glioblastoma multiforme (GBM) cells (GBMcs). This process is essential for inducing changes in the pericytes’ anti-tumoral and immunoreactive phenotypes. Starting from the initial stages of carcinogenesis in GBM, PCs conditioned by GBMcs undergo proliferation, acquire a pro-tumoral and immunosuppressive phenotype by expressing and secreting immunosuppressive molecules, and significantly hinder the activation of T cells, thereby facilitating tumor growth. Inhibiting the pericyte (PC) conditioning mechanisms in the GBM tumor microenvironment (TME) results in immunological activation and tumor disappearance. This underscores the pivotal role of PCs as a key cell in the TME, responsible for tumor-induced immunosuppression and enabling GBM cells to evade the immune system. Other cells within the TME, such as tumor-associated macrophages (TAMs) and microglia, have also been identified as contributors to this immunomodulation. In this paper, we will review the role of these three cell types in the immunosuppressive properties of the TME. Our conclusion is that the cellular heterogeneity of immunocompetent cells within the TME may lead to the misinterpretation of cellular lineage identification due to different reactive stages and the identification of PCs as TAMs. Consequently, novel therapies could be developed to disrupt GBM-PC interactions and/or PC conditioning through vascular co-option, thereby exposing GBMcs to the immune system.

## 1. Introduction

Gliomas are the most aggressive brain cancer. Glioblastoma multiforme (GBM; recently classified as grade IV astrocytoma by the World Health Organization) is a high-grade infiltrative neoplasm with a life expectancy of 15 months after diagnosis [1,2]. The poor prognosis for GBM is due to its cellular heterogeneity, high infiltration capacity in the brain parenchyma, and associated repression of immune system activation [3]. GBM is a relatively common brain tumor, with an incidence of 5–7 cases per 100,000 individuals [4]. Since the neurobiological mechanisms underlying GBM development and progression are incompletely understood, the established treatment, known as the Stupp protocol, still dates to 2005 [3] and includes surgical resection, radiotherapy, and chemotherapy with temozolomide (TMZ). Therefore, it seems urgent to intensify research to understand the neurobiological aspects underlying tumor development and progression in order to propose more efficient treatments to replace the Stupp protocol [5].

The cellular mechanisms that orchestrate glioma generation are only partially understood because the primary events underlying how primary cancer stem cells, once generated in the central nervous system, evade immunological surveillance have not been studied in human brain samples. This may be due to the difficulty in identifying glioblastoma stem cells during their primary interaction with cells in the brain parenchyma. Therefore, our current knowledge derives from experimental models that, in vitro or in vivo, have shed some light on the initial events of cancer generation. The accumulation of gene mutations in neural progenitors, leading to unregulated tumor cells which undergo malignant progression to cancer stem cells, is the prevailing theory that will be extensively described below. Additionally, studies on experimental carcinogenesis in animal models are still far from capturing the real complexity of the human brain. Thus, initial events after the transformation of neural progenitors into malignant glioma cells, such as GBM, have not been analyzed in the human context regarding the immunological repression required to evade natural immunological surveillance. In this review, we will focus on the main cellular mechanisms underlying the immunosuppressive activity of GBMcs on normal cells in the tumor microenvironment (TME), with emphasis on the role of brain pericytes (PCs) in the process of GBM infiltration. We consider that the infiltration front of GBM is a scenario that may recapitulate the primary interaction of tumor cells with normal PCs in the neurovascular unit.

## 2. The Role of Pericytes in Glioblastoma Multiforme Generation and Progression

**Figure 1:**
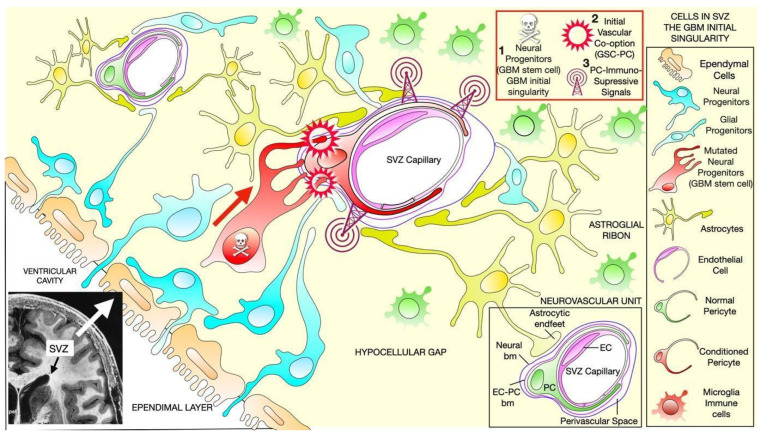
Schematic representation of initial cellular interactions in the tumor microenvironment (TME) at the subventricular zone (SVZ) and periventricular region of the brain (inset located in the lower left corner). Neural progenitors are in contact with the ependymal cell layer and migrate into the astroglial ribbon, where the first glioma stem cell (GSC) is generated due to the accumulation of oncogenic mutations. We refer to this primary event as the “glioblastoma multiforme initial singularity”. The GSC’s angiotrophism physically interacts with local pericytes (PCs), promoting vascular co-option and immunosuppressive activity in PCs. Additionally, glioma-conditioned PCs induce immunosuppressive characteristics in other immunocompetent cells: tumor-associated macrophages (TAMs) and microglia. Abbreviations: EC: endothelial cell; EC-PC bm: basal membrane of SVZ capillary; neural bm: neural parenchyma basal membrane; PC: pericyte; and SVZ capillary: lumen of the subventricular zone’s capillary.

**Figure 2:**
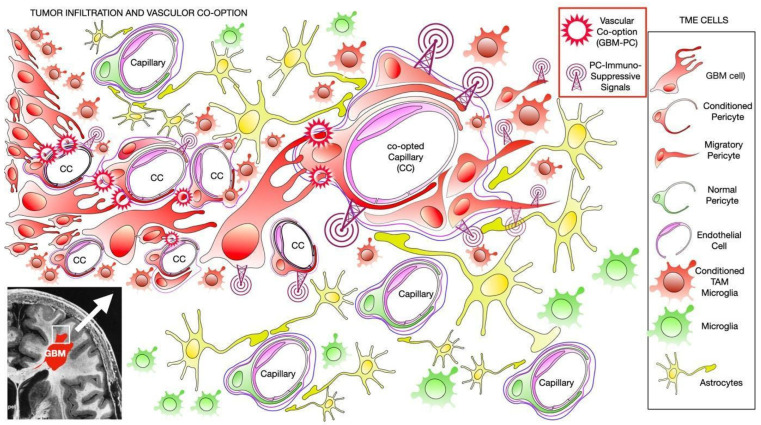
Schematic representation of the infiltrating front of glioblastoma in the frontal lobe of the left hemisphere (inset located in the lower left corner). Glioblastoma cells infiltrate the peritumoral parenchyma through perivascular spaces, establishing contact with pericytes (PCs). Glioma-conditioned pericytes (GBM-PCs) proliferate in the perivascular space and migrate outside the perivascular spaces, developing vascular co-option mechanisms such as capillary deformation and immunosuppression. Here, pericytes and tumor-associated macrophages (TAMs) share similar immunosuppressive phenotypes. Abbreviations: CC: glioblastoma-co-opted capillary; GBM: glioblastoma multiforme tumor mass; and GBM-PC: glioblastoma-co-opted pericyte.

Tumor progression and infiltration through the perivascular space are some of the most important characteristics of GBM, as they cause alterations to blood vessels in a process called vascular co-option, which is identified by vascular malformation at the infiltrative tumor edges [5,6]. Vascular co-option has been identified as a process required to activate the immunosuppressive properties of GBM [7]. Since the brain is a highly vascularized structure, the angiotropism of GBMcs favors, first, tumor generation by repressing immune system activation against cancer stem cells and, second, tumor expansion by maintaining immunosuppression and controlling inflammatory signals.The initial stages of cancer development, which we can refer to as the “GBM initial singularity”, entail mutations occurring in oncogenic genes within neural precursors, leading to the generation of GBM stem cells (GSCs; [8]). Undifferentiated neural precursors may represent the origin of GSCs since single-cell RNA sequencing has identified tumor-intrinsic transcriptional signatures of both neuronal and glial progenitors [9]. This occurs in the densely vascularized subventricular zone (SVZ) of the brain, underneath the ventricular epithelium of the lateral ventricles [10]. Therefore, primary-generated GSCs are in contact with the extracellular matrix and the microenvironment-resident cells in the perivascular spaces of the SVZ capillary network. This is where the initial vascular co-option will provide perivascular cell-to-cell interactions that will underlie the cellular and molecular mechanisms of how GSCs evade the immune system (Figure 1). Once the tumor is established, these mechanisms are recapitulated when GBMcs proliferate and infiltrate into the normal brain parenchyma, migrating through perivascular spaces and contacting PCs [7]. Consequently, GSCs generate the tumor by co-option in pre-existing vasculature, which activates and maintains immunosuppressive induction in TME, and it is utilized as a scaffold to migrate into the normal brain stroma between vessels [7,10]. We have demonstrated that the principal target cell of GBM during vascular co-option is the pericyte [7,11], which constitutes the primary cell type in the normal brain perivascular space. Then, cell-to-cell contact between GBMcs and PCs, along with the subsequent activation of pro-tumoral cellular and molecular processes in PCs, is essential for GBM growth and progression (Figure 2) ([7,11,12] and reviewed in [13]). Additionally, following primary vascular co-option after GBM initial singularity, the GBM is generated and will invade the surrounding parenchyma, with the subsequent alteration of the brain–blood barrier (BBB), allowing other cells such as tumor-associated macrophages (TAMs) to infiltrate perivascular spaces, as well as the activation of microglia in the tumor microenvironment (TME) [14,15]. These cells will also contribute to cancer cells’ ability to evade the immune system, leading to a poor prognosis. Notably, under conditions of BBB disruption, there appears to be a significant contribution of bone marrow-derived macrophages to the brain macrophage pool in a pathological context [16]. Since, in brain tumors such as gliomas, the rupture of the BBB occurs with disease progression [17], at the very initial generation of the first GSCs (GBM initial singularity), the cellular impermeability of the BBB should not be impaired. Therefore, the immunocompetent pool of cells in the TME is exclusively represented by PCs.

## 3. Cellular Immunity in Glioblastoma Multiforme

The immune system’s role in fighting cancer involves identifying and destroying abnormal cells expressing cancer-specific antigens, but cellular and molecular mechanisms that can hinder this process have been described in cancer. For instance, in solid tumors, cancer cells exhibit strong molecular heterogeneity that, in GBM, is progressively increased by its characteristic genetic instability, making it complex to develop specific immunological responses to the entire cancer cell population. We have described how cancer cells closely interact with non-tumoral cells in the tumor microenvironment (TME), which play a fundamental role in regulating immunological responses [7,11,12]. Glioblastoma cells create an immunosuppressive TME at the edges of the tumor, where they encounter normal brain parenchyma cells, protecting GBMcs from detection and elimination by the immune system. They can release immunoregulative factors and establish cellular interactions that suppress immune responses, allowing the tumor to evade detection and destruction by immune cells [11,12,13,14]. Communication between GBMcs and TME cells is achieved through exosome interchange, soluble factors such as cytokines, chemokines, matrix-remodeling enzymes, and growth factors. Additionally, it has been demonstrated that GBMcs employ cell-to-cell contact-dependent signals, including filopodia, intercellular gap junctions, and tunneling nanotubes (reviewed in [13,15]). Therefore, it is fundamental to understand the processes activated by the communication between GBMcs and normal cells in the TME for GBM initiation and progression, as well as for better targeting GBM therapeutically (reviewed in [16]).

Although activation immunity seems like a promising therapy in GBM, various strategies to harness the immune system’s power to fight glioblastoma, which involve using drugs to stimulate the immune system to recognize and attack cancer cells, have not yielded efficient results so far. For example, therapies like immune checkpoint inhibitors aim to overcome the tumor’s immunosuppressive ability, and vaccines that recognize specific markers on glioblastoma cells or utilize engineered immunocompetent cells (CAR-T cells) to target the tumor initially promised success in the preclinical and initial phase of clinical trials but have not demonstrated therapeutic efficiency in phase III clinical trials or approved therapies. In a recent review, Losurdo et al. [17] identified 130 articles describing clinical trials using strategies to block GBM’s immunosuppressive properties, showing that important efforts have been made, but without enough significant and efficient results to progress beyond the Stupp protocol. Therefore, new elements must be incorporated into the understanding of GBM’s immunologic mechanisms to develop targeted and effective new therapies.

Given that the immunotolerance of cancer may be initiated by interactions between GBMcs and PCs [11,12,13], which commence during the early stages of tumor development subsequent to the initial singularity of GBM in the hypervascularized SVZ, the conditioning of PCs by GBMcs in the process of vascular co-option, conferring an immunosuppressive state in the TME, may strongly diminish or impede the efficiency of immunological stimulation-dependent therapies. Therefore, these immunotherapies, which are specifically targeted at cancer or other cells within the TME, have little chance to have success without previously suppress PCs’ immunoregulatory activity. Hence, the interplay between GSCs and PCs is pivotal in promoting immunotolerance, allowing the tumor to evade immune surveillance and, therefore, proliferate to generate a tumor mass and progressively grow and infiltrate peritumoral parenchyma. The GBM tumor microenvironment is highly immunosuppressive, creating a shield against immune attacks even after immunotherapies.

We have described previously how the microenvironment of gliomas, which encompasses various cell types and the extracellular matrix surrounding the tumor, plays a crucial role in tumor progression and may determine the therapy response. According to Sharma et al. [16], cells within the TME, such as T cells, mast cells, tumor-associated macrophages (TAMs), cancer-associated fibroblasts (CAFs), and natural killer (NK) cells, could elicit a robust immune response against the tumor.

We are discussing some cellular characteristics within the glioma and tumor microenvironment that are closely related to GBM immunotolerance properties.

### 3.1. Cells in Glioma TME

#### 3.1.1. Tumor Cells

As previously described, gliomas originate in the SVZ from neural precursors [8,9]. With the proliferation of GSCs, mutations accumulate in these cells, resulting in high intratumor cellular heterogeneity characterized by the presence of multiple cell populations in a single tumor mass. GSCs proliferation is potentiated by direct interaction with PCs [7,11]. It has been demonstrated that the presence of GSCs with self-renewing and multi-lineage differentiation properties is the cause of tumor initiation, growth, and recurrence during the progression of GBM [18]. Cellular heterogeneity in GBM enables responses to different normal brain niches and therapeutic pressures, contributing to tumor aggressiveness, growth, and treatment failure. For instance, intrinsic cellular and molecular expression profiles determine the classification of glioblastoma into three clusters: classical (CL), proneural (PN), and mesenchymal (MS). Recently, a new cluster has been identified as the glioma-CpG island methylator phenotype (G-CIMP) [19]. These molecular subtypes of GBM correspond to a characteristic tumor histopathology, but, due to tumor cell heterogeneity, they may be detected in different areas of the same tumor mass. Although specific genetic alterations have been associated with these molecular subtypes, *EGFR* gene alterations to CL, TP53 and *PDGFRA* gene alterations PN, and *NF1* gene alterations to MS, the MS profile appears to represent a worsening evolution of the other three [20]. However, intratumor heterogeneity complicates current efforts to detect molecular biomarkers that must be homogeneously distributed in the tumor cell mass to be identified as efficient therapeutic targets, necessitating an individualized approach to implement personalized medicine. Thus, “omic” technologies to stratify patients and a combination of different therapies to target patient-specific molecular and cellular aberrations appear necessary to improve therapeutic efficacy and prognosis.

#### 3.1.2. Immune Cells

Both innate and adaptive immune cells infiltrate gliomas. These include microglia, macrophages, T cells, B cells, and natural killer cells. We have described previously that PCs are immunocompetent cells present in the capillary bed that play a fundamental role in activating the immune response, in addition to being consistently present in the TME, without the requirement of migrating or infiltrating from other compartments. The interaction between these immune cells and tumor cells can strongly influence the progression and response to treatment.

Immunosuppressive Cells: Several types of immunosuppressive cells such as pericytes (PCs), tumor-associated macrophages (TAMs), and M2-like microglia are found in the TME.

1. Pericytes

We have already described previously that cell–cell contact between tumor cells and PCs alters the behavior of PCs, leading to the activation of various mechanisms involved in vascular co-option, including the formation of peritumoral vascular malformations in infiltration areas and the regulation of the immunologic response. Although the roles of PCs in the brain TME are not yet fully understood, our recent studies have demonstrated that direct interaction between PCs and GBMcs is essential to trigger alterations in the immune phenotype of PCs [11,12]. From the initial stages of GBM development, GBM-conditioned PCs acquire an immunosuppressive phenotype characterized by the secretion of high levels of anti-inflammatory cytokines (IL10, IL6, TGF-β), the expression of immunosuppressive molecules such as programmed death-ligand 1 (PDL-1), and the reduced expression of co-stimulatory molecules (IL2). Additionally, they exhibit a significantly impaired capacity to activate competent T cells, thereby promoting tumor growth. Our research has demonstrated that the interaction between GBMcs and PCs induces chaperone-mediated autophagy (CMA) in PCs [11,12]. CMA is a lysosomal process that selectively degrades intracellular proteins [21]. The chaperone–substrate complex binds to lysosome-associated membrane protein type 2A (LAMP-2A), and the substrate protein then unfolds with the aid of chaperones. Therefore, LAMP-2A expression and CMA are critical for maintaining cell function and homeostasis by selectively degrading proteins and modulating their response to various stimuli [22].

LAMP-2A expression is up-regulated specifically in PCs contacted by GBMcs in co-culture experiments of mouse PCs with human GBM cell lines, animal models of human GBM xenotransplants in immunocompetent mice, and GBM biopsies from patients [11,12]. We have demonstrated that immune system activation is mediated by PCs and is inhibited when GBM induces CMA in these cells after vascular co-option. Immune reaction against GBM is restored when CMA is impaired in mouse PCs, both in in vitro and in vivo experimental models [12]. Since CMA depends directly on the levels of LAMP-2A at the lysosomal membrane [23,24], PCs derived from Lamp-2a knockout mice, when in contact with GBMcs, exhibit reduced CMA activity, leading to the up-regulation of immune and inflammatory responses in vivo, expressing and secreting anti-tumoral signals, along with the downregulation of genes related to the pro-tumoral phenotype in vitro. These findings are consistent with the anti-tumoral behavior of PCs observed when CMA is inhibited [12,25,26]. Thus, the activation of CMA due to the interaction between tumor cells and PCs alters the PC-secreted proteins (secretome) to an immunotolerant profile rich in factors that suppress the immune response and hinder tumor clearance [25,26]. Molina et al. [25] analyzed the differential expression of secretome from control PCs or PCs with ablated autophagy (Lamp-2a knockout) in co-cultures with GBMcs. The secretome of normal PCs in GBM co-cultures differed from that of PCs with ablated autophagy, with differences in the cell adhesion proteins implicated in cytoskeleton dysregulation, leading to defects in adhesion and motility. This alteration in PCs’ cellular contacting properties is especially relevant because specific contacts between cytotoxic lymphocytes (T cells) and PCs, as antigen-presenting cells, known as immunological synapses, are essential for stimulating immune responses. The increased expression of inhibitory ligands in cancer causes the accumulation of actin around these immunological synapses, inhibiting proper immune function [27]. Conversely, a comparative analysis of the secretome of normal PCs and PCs with ablated autophagy co-cultured with GBM cells revealed the differential expression of several anti-tumor proteins, including secreted proteins related to tumor apoptosis and anti-angiogenesis [25]. While the co-culture of GBMcs with PCs increases the proliferation rate of cancer cells compared to GBM cultures alone, LAMP-2a knockout PCs, which cannot be conditioned by GBMcs because of the deficient intercellular stabilization of GBMcs contacts with PCs, can even eliminate tumor cells in vitro, indicating direct anti-tumoral properties in their secretome [11,14]. Thus, this analysis using Lamp-2a knockout PCs shows that cell–cell contact between GBMcs and PCs is a crucial factor in promoting tumor progression through vascular co-option, in agreement with our previous results [7], as well as the fact that changes in the PC secretome underlie the processes which generate an immunosuppressive microenvironment by inducing aberrant CMA in PCs. This subsequently leads to changes in the actin cytoskeleton, likely affecting pericyte–T cell interactions.

Birbrair et al. [28] described two types of PCs regarding their molecular and functional characteristics in oncologic experiments. While Type 1 PCs (Nestin-/NG2+) do not have infiltrative or angiogenic activity, Type 2 PCs (Nestin+/NG2+) infiltrate and participate in brain tumor neovascularization. In our experiments, although we demonstrated that the PCs were NG2+, we did not specifically determine the expression of Nestin. But the detection of angiogenic signals’ over-expression in GBM-conditioned PCs and its downregulation in Lamp2a knockout PCs supported the notion that the PCs that we analyzed were Type 2 [25]. The important role of GBM-conditioned PCs in GBM generation and progression has been further demonstrated in other studies by the increased tumor growth observed in xenotransplants of GBMcs together with PCs compared to GBMcs xenotransplants alone [17]. Moreover, in established tumors following human xenotransplants in immunocompetent mice, the intracerebral or intravenous transplantation of Lamp-2a knockout PCs resulted in the elimination of glioblastoma with the activation of immune and inflammatory mechanisms [25]. Thus, the reduction in pericytes in brain capillaries may represent protection against gliomas because these helper cells are absent to evade the immunological reaction. This pro-tumoral role of PCs is also supported by the generation of tumor-derived PC-like cells due to the transdifferentiation of GSCs into PCs, which increases cancer-induced angiogenesis and tumor growth [29]. Interestingly, transforming growth factor β (TGF-β) induces the differentiation of GSCs into PCs. Given that we have demonstrated an over-expression of TGF-β in GBM xenotransplants, in coincidence with co-grafted PCs localization [11], there may be a potential reinforcement of tumoral-derived PC generation in relation to vascular co-option in the infiltration areas.

In a recent publication, Hoogstrate et al. [30] studied the molecular evolution of IDH-wild-type glioblastoma and observed that the tumor cell signature remained stable throughout tumor evolution. They found that the mesenchymal subtype of GBM was associated with a lower survival compared to the other two subtypes, in agreement to Bowman et al. [20]. The increase in the mesenchymal omics’ subtype signature over time was attributed by these authors to PC transcriptome modification, probably as a consequence of GBMcs-PCs interaction. This suggests that the increasing presence of PCs conditioned by GBM cells may lead to glioma evolution towards the mesenchymal subtype and worsen the prognosis.

Pericyte loss has been reported in Alzheimer’s disease (AD) even at the initial stages of dementia [31,32,33,34], as part of the associated vasculopathy of neurodegeneration. This pericyte loss was localized in the brain white matter, cortex, hippocampus, and retina of patients with AD (reviewed in [34]). Furthermore, PCs deficiency in humans and mouse models accelerates BBB breakdown, alterations in PCs–endothelium crosstalk, and neuroinflammation in AD and vascular dementia (reviewed in [35]). This suggests that PCs depletion might contribute to the described inverse comorbidity between AD and GBM. In a recent letter to the editor published in the *Medical Oncology* journal, Mokbul and Siddik [36] reviewed the data both supporting and refuting this indirect comorbidity. Their conclusion was that, although both conditions are linked with aging, several challenges hinder accurate epidemiological studies: (1) the heterogeneity of studies, which complicates comparative analysis; (2) the low survival rates among patients with GBM, making it challenging to assess the incidence of AD in patients diagnosed with GBM; and (3) the identification of common molecular mechanisms that may act in different directions, either promoting or hindering comorbidity. A reduction in PCs in AD because of the neurodegenerative process has been documented [34,35], potentially providing protection against GBM development due to increased difficulty in primary vascular co-option and PC-mediated immunological conditioning, which are necessary for GSCs’ evasion of the immune system.

Another disease in which pericytes are reduced is diabetes mellitus (DM) [34]. In a comprehensive meta-analysis aimed at clarifying the association between DM and the risk of gliomas, Zhao et al. [37] analyzed data from seven case–control and four cohort studies involving more than 580,000 individuals. The results obtained from the case–control studies suggested that DM was significantly associated with a decreased risk of gliomas in Caucasian patients. There were no studies involving Asian patients or other races. In a subgroup analysis by gender, the inverse association between DM and gliomas was more apparent in men than in women. Although they interpreted this inverse comorbidity as being caused by gender-specific hormonal changes in patients with diabetes, pericyte depletion may also represent an important protection factor for developing GBM due to the increased difficulty in primary vascular co-option.

Montagne et al. [38] studied the reduction alteration in PCs in the human hippocampus during normal brain aging. Contrary to the data observed in AD and DM, these results contradict the increasing incidence of GBM with age. The median age of diagnosis for patients with GBM is 64 years old, with the incidence increasing for patients between 75 and 85 [39]. Therefore, we must consider additional factors contributing to brain aging, such as the initial heightened permeability of the blood–brain barrier (BBB). This increased permeability allows macrophages with immunoregulatory properties to leak out during the early stages of GBM, contributing to the formation of a TME which suppresses the immune system. This could potentially represent a compensatory mechanism in response to PCs reduction, thereby initiating primary immunoregulation independent of initial vascular co-option. Moreover, as we have described before, the molecular mechanisms of action in aging and GBM generation may act in different directions, either promoting or hindering comorbidity [35]. This dynamic may explain the positive correlation between GBM and aging.

2. Tumor-Associated Macrophages (TAMs)

The tumor microenvironment of GBM can contain more than 80% of tumor-associated macrophages (TAMs) and microglia [40,41]. TAMs have the capability to release cytokines and growth factors that facilitate tumor proliferation, survival, and metastasis progression, while also impeding the function of immune cells. During the early stages of cancer development, immune cells and tissue-resident macrophages are expected to attach to cancer cells by creating pro-inflammatory conditions and presenting phagocytosed antigens for protection against GBM development. This protection should imply a difficulty in primary vascular co-option and immunological conditioning of PCs, cells which, as we have described before, are required for GSCs to evade the immune system, activating their major histocompatibility complex (MHC) class II (M2) receptors [11]. This leads to the infiltration of anti-tumor M1 macrophages, CD8+ T cells, and natural killer cells, which maintain an inflammatory secretome against cancer cells and provide chemotactic signals to replace these immunocompetent cellular populations [42,43,44,45]. Bikfalvi et al. [46] reviewed the molecular and cellular mechanisms of the tumor microenvironment involved in the polarization of anti-tumor macrophages (M1) into pro-tumor macrophages (M2), in the context of primary cancer development and metastasis. In GBM, excluding PCs, TAMs and microglia are the most abundant immunocompetent cells that invade tumors and may initiate M1 anti-tumoral processes [42]. However, in GBM, these cells are polarized towards an immunosuppressive (M2) phenotype, promoting tumor growth and suppressing immune responses. Therefore, TAMs are categorized as either M1- or M2-polarized cells, exhibiting relative pro-inflammatory/anti-tumor or anti-inflammatory/pro-tumor properties, respectively. Curiously, these two polarized states can transition from one to the other. TAMs’ heterogeneity in the TME has been extensively studied, and the interaction of TAMs with PCs has been described but exclusively in relation to tumor-induced angiogenesis [47]. However, it appears evident that the transition from the M1 to M2 macrophagic phenotype in TAMs and microglia, namely, from immunoreactive to immunosuppressive phenotypes, is not a spontaneous transformation. Rather, it must be induced from the outset signals in cancer genesis. Apart from the hypothetical direct primary influence of GSCs on other resident cells, PCs may play a pivotal role in this process, as demonstrated in previous research [11,12,14,17,48]. In the production of anti-inflammatory cytokines, such as IL10, IL6, and TGF-β, by GBM conditions, PCs have been postulated as the mechanism to hinder the function of other antigen-presenting cells [11]. There are significant research efforts focused on understanding the mechanisms underlying TAMs’ immunosuppressive activity in the TME, because this process has been identified as a key element in overcoming the problem of the reduced efficacy of immunotherapy against GBM. The highlighted significance of TAMs in GBM-specific tumor immunotolerance has not encompassed PCs in the mechanisms of GBM immunosuppression, underscoring an incomplete comprehension of this fundamental mechanism in GBM biology.

3. M2-like Microglia

These specialized brain-resident immune cells can adopt an immunosuppressive M2-like phenotype like PC and TAM, thus facilitating tumor progression [44].

Data from single-cell RNA sequencing technology have been reviewed by Khan et al. [45], elucidating the cellular and molecular heterogeneity of TAMs and microglia. This molecular heterogeneity observed in immunocompetent cells within the TME may represent distinct reactive states resulting from the interaction between GBMcs and TME cells. Given the absence of definitive and reliable markers for specific immunocompetent cells within the TME, it is plausible to consider that conditioned PCs could potentially be identified as TAMs or reactive microglia, particularly during the early stages of GBM initiation and at the infiltration edges where vascular co-option predominates and where both PC proliferation and migration have been observed [11,12,13].

### 3.2. Origin of Cells in Glioma TME

Bowman et al. [20] describe distinct expression profiles in brain microglia and recruited bone marrow macrophages associated with tumor-mediated effects. Notably, these profiles are influenced by chromatin landscapes established prior to tumor initiation. Pericytes are the cells that influence this pro-tumoral landscape in the subventricular zone (SVZ), where neural and glial precursors undergo glioma-related mutations and generate GSCs [37]. The neural crest origin of brain PCs is an ontogenetic singularity [49]. They infiltrate alongside endothelial cells into the embryonic brain parenchyma, generating, with endothelial precursors, the neurovascular unit and Virchow–Robin spaces [50]. This pattern of origin and distribution of PCs closely coincides with TAMs from perivascular macrophages in the brain [51]. Since the cephalic bone marrow, which provides immunocompetent cells to cephalic organs, is derived from neural crest-derived skull bones [52], we can hypothesize that the first macrophages in the TME may be derived from skull bone marrow, entering the meninges and cerebrospinal fluid in the subarachnoid space and then penetrating into the brain parenchyma through Virchow–Robin spaces in contact with PCs. In this scenario, skull-derived macrophages may penetrate the initial tumor mass before blood–brain barrier (BBB) disruption and interact with PCs at the onset of GBM formation. This may represent a new way that needs specific effort to explore the relation between PCs and primary macrophages in the perivascular spaces of the brain. Subsequently, in infiltrating tumor zones, both skull- and vertebral bone marrow-derived macrophages may enter the TME through the permeable BBB.

The possibility of identifying conditioned PCs as TAMs is supported by direct experimental studies demonstrating that, in ischemic brain models, PCs can differentiate into macrophage-like cells. This is evidenced by the expression of molecular profiles and the presence of a CD45-high CD11b+ macrophagic-specific phenotype in PCs [20,53]. Furthermore, achieving precise specificity in the selection of immunocompetent cells exclusively within the TME is not a straightforward process, as cell selection for macrophages has been based on the expression of CD49d, a glycoprotein integrin subunit also expressed in PCs [20,54], as well as other specific macrophage markers expressed in PCs after various neurotoxic stimuli [55].

## 4. Therapeutic Challenges

The immunosuppressive microenvironment presents significant challenges for immunotherapies, such as checkpoint inhibitors and adoptive cell therapies, as they encounter difficulty in overcoming these inhibitory signals to mount an effective anti-tumor immune response. Understanding and targeting these mechanisms within the glioblastoma microenvironment is crucial for developing effective immunotherapies that can overcome immunotolerance and improve patient treatment outcomes [56,57,58]. The future of immunotherapy for GBM requires a collaborative approach, integrating rational combinations of vaccine therapy, cell therapy, as well as radio- and chemotherapy, alongside targeted molecular therapy focused on the tumor microenvironment.

### Immunotherapy

Immunotherapy aims to harness the body’s immune system to recognize and attack cancer cells. GBM tumors often face challenges because, as we have extensively described in this review, they can develop in an environment that evades the immune system. Immunotolerance, a process during which the immune system fails to recognize cancer cells as threats, is a significant hurdle in treating these brain tumors.

Several approaches attempt to overcome immunotolerance in GBM (reviewed in [16,41,59,60]):

1. Immune Checkpoint Inhibitors: These drugs target proteins that inhibit the immune response, such as PD-1 or CTLA-4. By blocking these proteins, immune cells can recognize and attack cancer cells more effectively.
2. Therapeutic vaccines are designed to train the immune system to recognize specific antigens found on glioma cells. These vaccines stimulate an immune response against the tumor.
3. Chimeric Antigen Receptor (CAR) T Cell Therapy: This involves modifying a patient’s own T cells to recognize specific antigens on glioma cells. The modified T cells are then reintroduced into the patient to target and destroy the tumor cells.
4. Tumor-Infiltrating Lymphocytes (TILs): TIL therapy involves extracting immune cells from the tumor, selecting the ones that recognize the tumor, multiplying them in the lab, and infusing them back into the patient.
5. Cytokine Therapy: This involves using cytokines, which are small proteins involved in cell signaling, to stimulate the immune system to attack cancer cells.

However, challenges persist. The brain possesses a distinct immune environment due to the BBB, which can impede the efficacy of certain immunotherapies. Concerning systemic cellular therapy, it is crucial to acknowledge that PCs play a significant role as structural cells in maintaining the functionality of the BBB. Thus, they occupy a privileged position to immunosuppress immune-modified cells promptly upon vascular extravasation or diffusion through cerebrospinal fluid. Furthermore, as previously mentioned, GBMcs exhibit genetic diversity and heterogeneous antigenic profiles, rendering the tumor mass less responsive to targeted treatments.

The interaction between GBMcs and TME cell types is complex. As a consequence of this interaction, GBM creates an immunosuppressive microenvironment that hampers the body’s natural immune response, allowing tumors to evade destruction. Strategies targeting the microenvironment, such as modulating immune cell activity, are being explored to enhance the effectiveness of therapies against GBM. Perivascular PCs are fundamental in initiating immunotolerance mechanisms, and, therefore, the interaction between GBMcs and PCs should be regarded as a crucial target for developing new therapies.

Recently, Mendez-Gomez et al. [61] created ‘‘onion-like’’ multi-lamellar RNA lipid particle aggregates (RNA-LPAs) to enhance the immunogenicity of tumor mRNA antigens (synthetic glioma-associated antigens and whole-tumor mRNA). RNA-LPAs reprogrammed the TME towards immunoreaction in GBM animal models and in a phase I human trial with three patients with GBM, detecting increased cytokine/chemokine release and immune activation in the TME. 

## 5. Strategies for Targeting GBM–Pericyte Interactions

The physical interactions between GBMcs and PCs are established through the production of cellular filopodia or flectopodia and nanotubules. These structures are normal cellular mechanisms required for GBM cell migration. [12,47,62]. We have previously described how flectopodia/nanotubules-mediated physical interaction between GBMcs and PCs induces changes in the PCs’ response to tumor cells, which underlie vascular co-option. This process mediates capillary malformation, as well as alterations in the PCs’ transcriptome and secretome, leading to the development of an immunosuppressive phenotype which promotes tumor immunotolerance. The activation of the CMA is the basic mechanism in this process because, after blocking the CMA in PCs, the interaction with GBMcs is disrupted and immunosuppression is not activated; then, the non-conditioned PC transcriptome and secretome show anti-tumoral properties, and the tumor is eliminated [7,11,12,25]. Therefore, identifying PCs as therapeutic targets may be a possibility through different approaches, such as tracking tumor progression by selectively radioactively labeling PCs, blocking pericyte proliferation, as well as blocking PC–GBMc interactions via Cdc42 inhibition [63,64]. Selectively tumor-conditioned PCs could represent a promising therapeutic strategy for GBM. However, healthy PCs must be protected since they are fundamental to the immune response to tumor generation and progression. In fact, it has been shown that the elimination of PCs in experimental tumors did not improve anti-tumoral treatments, decreasing the immune response to cancer cells and emphasizing the importance of PCs in the immunological response against cancer development [63,64]. Therefore, therapeutic strategies targeting GBM-PC contacts could represent a more effective anti-tumoral approach. Since Cdc42 is required for co-option and flectopodia formation, blocking Cdc42 activation or blocking Cdc42 gene production represents an interesting strategy for preventing GBM-PC cell contact and PC corruption. As a promising example, ARN22089 is a molecule that blocks the interaction between Cdc42 GTPases and their effectors in mouse melanoma models and in patient-derived xenografts in vivo [64].

Moreover, we have shown that the increase in CMA in GBM-conditioned PCs underlies PC transformation and flectopodia stabilization [11,12,25]. Therefore, CMA regulation could be another therapeutic target in PCs around the tumoral mass. While CMA activity is greater in cancer cells and pericytes, it would seem that the anti- or pro-cancer function of CMA depends on tumor cell transformation and the interaction with local PCs. In addition, the significance of the homeostatic role of CMA in cells, which involves multiple intracellular signals and proteostasis, determines the importance of directed research into context-dependent therapies [65].

Further studies are needed to fully understand the cellular and molecular mechanisms underlying GBM-PC communication and the changes in PC behavior towards helper cells in glioblastoma generation after GBM initial singularity in favor of GSCs, as well as in cancer cells’ proliferation and GBM infiltration. By doing so, we may move closer towards developing a definitive treatment for this devastating disease.
